# Fast Multispectral Optoacoustic Tomography (MSOT) for Dynamic Imaging of Pharmacokinetics and Biodistribution in Multiple Organs

**DOI:** 10.1371/journal.pone.0030491

**Published:** 2012-01-25

**Authors:** Adrian Taruttis, Stefan Morscher, Neal C. Burton, Daniel Razansky, Vasilis Ntziachristos

**Affiliations:** Institute for Biological and Medical Imaging, Helmholtz Zentrum Munich, Technical University Munich, Munich, Germany; University of Queensland, Australia

## Abstract

The characterization of pharmacokinetic and biodistribution profiles is an essential step in the development process of new candidate drugs or imaging agents. Simultaneously, the assessment of organ function related to the uptake and clearance of drugs is of great importance. To this end, we demonstrate an imaging platform capable of high-rate characterization of the dynamics of fluorescent agents in multiple organs using multispectral optoacoustic tomography (MSOT). A spatial resolution of approximately 150 µm through mouse cross-sections allowed us to image blood vessels, the kidneys, the liver and the gall bladder. In particular, MSOT was employed to characterize the removal of indocyanine green from the systemic circulation and its time-resolved uptake in the liver and gallbladder. Furthermore, it was possible to track the uptake of a carboxylate dye in separate regions of the kidneys. The results demonstrate the acquisition of agent concentration metrics at rates of 10 samples per second at a single wavelength and 17 s per multispectral sample with 10 signal averages at each of 5 wavelengths. Overall, such imaging performance introduces previously undocumented capabilities of fast, high resolution *in vivo* imaging of the fate of optical agents for drug discovery and basic biological research.

## Introduction

Macroscopic near-infrared fluorescence (NIRF) imaging has been widely applied to studies of biodistribution of various optical agents in basic biological and pharmaceutical research [1], enabled by the low tissue absorption of light in the near-infrared (NIR) wavelength region and the sensitivity of fluorescence detection. Fluorescent agents vary from fluorescent proteins [2] through a multitude of targeted agents [3], to enzyme-activatable fluorescent probes [4], [5]. However, macroscopic NIRF imaging has limitations. Simplistic epi-illumination implementations produce surface-weighted images that do not accurately reveal deep-tissue activity [6]. Tomographic implementations produce quantitative, volumetric results, but at the expense of image acquisition times in the range of tens of minutes, unsuitable for capturing fast-changing signals [7]. In any case, the spatial resolution of purely optical methods degrades rapidly with imaging depth: whole-mouse optical imaging generally results in resolutions in the order of millimeters [6].

It is the high scattering of light in tissues that degrades spatial resolutions in optical imaging at increasing depths. This barrier can, however, be overcome by adding ultrasound detection to optical excitation in exploitation of the photoacoustic effect; that is, using optoacoustic imaging [8], [9]. Here, diffuse light heats local absorbers in the tissue, causing thermal expansion and giving rise to pressure waves, which can be detected by ultrasound transducers on the animal's skin surface. Suitable image reconstruction techniques then result in a spatial map of the absorbed energy [10]. By parallel detection using a multi-element ultrasound transducer array [11], [12], [13], real-time imaging capabilities as known from ultrasound imaging can be achieved, allowing the fast imaging we present here.

By using multiple excitation wavelengths, we resolve specific sources of absorption, whether tissue-intrinsic, like hemoglobin, or exogenous imaging agents (e.g. fluorescent dyes), by means of uniquely identifying their spectral absorption signatures [14]. Multispectral optoacoustic tomography (MSOT) has previously been applied to specific imaging of fluorescent proteins in model organisms [15], dye-enhanced kidney vascularization in mice [11] and targeted fluorescent agents and blood oxygenation in mouse brain tumors [16]. In addition to the detection of fluorescent dyes, optoacoustic techniques are uniquely capable of visualizing other light-absorbing materials, in particular those with absorption in the near-infrared, for example, gold nanorods [13] or carbon nanotubes [17].

We present MSOT as an *in vivo* imaging tool in studies of pharmacokinetics and biodistribution. To this end we imaged, in mice, with high temporal resolution, the disappearance rate of the near-infrared dye indocyanine green (ICG) from the circulation and its time-resolved uptake in the liver and gallbladder. Additionally, to demonstrate imaging of renal clearance, we visualized the uptake of a carboxylated fluorescent dye in different regions of the kidney. We extracted concentration-based metrics from our imaging data that showed the time-resolved signal curves in particular regions of interest.

## Materials and Methods

### Ethics statement

Procedures involving animals and their care were conducted in conformity with institutional guidelines and with approval from the Government of Upper Bavaria (agreement number 55.2-1-54-2531-64-08).

### Imaging Agents

**Figure 1:**
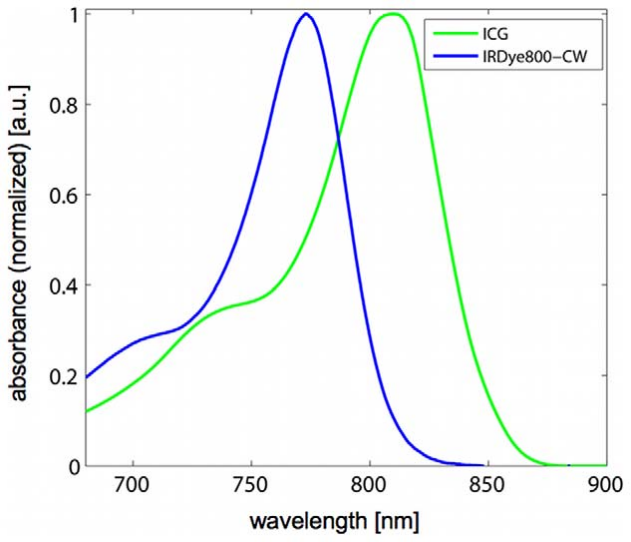
Absorption spectra of fluorescent agents ICG with albumin (green) and IRDye800-CW as measured in a spectrometer.

ICG (Pulsion Medical Systems, Germany) was selected due to its well-studied characteristics. It is an FDA approved, water-soluble, inert anionic tricarbocyanine dye that has been established as a tool to investigate a variety of different clinical endpoints such as hepatic function [18]. IRDye800-CW (Li-Cor) carboxylate is a near infrared dye that is water soluble and is rapidly excreted, unmetabolized, to a large extent by the kidneys [19]. The absorption spectra of the utilized agents (Fig. 1) show peaks in the near-infrared wavelength range.

40 nmol ICG and 20 nmol IRDye800-CW were diluted in saline and injected intravenously in a total volume of 100 µl to achieve final blood concentrations of approximately 20 µM and 10 µM, respectively.

### Experimental MSOT imaging system

We employed an experimental MSOT setup that has been described elsewhere in detail [11], [13]. It is capable of acquiring, reconstructing and displaying cross-sectional images through mice at a rate of 10 frames-per-second. Excitation in the near-infrared (700 nm–950 nm) is provided by a tunable optical parametric oscillator (OPO) pumped by an Nd:YAG laser (Opotek Inc., Carlsbad, CA). The laser pulse duration is below 10 ns and the pulse repetition frequency is 10 Hz. Light is coupled into a custom fiber bundle (CeramOptic Industries, Inc., East Longmeadow, MA) that is divided into 10 output arms, which serve to illuminate the mouse from multiple angles on the imaging plane. A custom-made piezocomposite ultrasonic transducer array (Imasonic SAS, Voray, France) with 64 elements and a central frequency of 5 MHz is used for detection. The elements are arranged in one row forming a spherical concave array covering 172° with a mechanical focal distance of 4 cm. The dimensions of the transducer array allow it to be considered as being cylindrically focused on one cross-sectional slice. A custom-built acquisition system with a total of 64 channels, a sampling rate of 40 million samples per second and 12 bit digital resolution records the time-resolved optoacoustic signals. Both the transducer array and fiber outputs are submerged in a water bath. Mice are placed in a horizontal position in a holder with a thin polyethylene membrane to prevent direct contact with water and allow acoustic coupling between mouse and transducer array. The laser beams and ultrasonic transducer array are in fixed position for all data acquisitions, whereas the mouse can be translated through the imaging plane using a linear stage (IAI Industrieroboter GmbH, Germany) to enable imaging of multiple transverse slices.

### Image reconstruction

Images were reconstructed using either a backprojection formula [8], which is particularly useful for fast reconstructions displayed on the imaging system during measurements, or a model-based approach [10] for offline analysis.

### Spectral unmixing

After image reconstruction, linear spectral unmixing was applied to resolve signals from ICG [20]. For each pixel in the image, the method fits the total measured optoacoustic spectrum to the known absorption spectra of the dye and oxy- and deoxyhemoglobin, which are expected to be the dominant absorbers in biological tissue. The fitting is performed using least-squares on the set of linear equations resulting from the multispectral measurements (1 equation per wavelength measured). The initial pressure distribution that underlies optoacoustic images is proportional to local light fluence in addition to the absorption properties, i.e.:
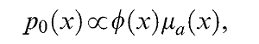
where *p_0_* denotes the initial pressure at a point in space *x*, *ϕ* is the fluence in Jm^−2^ and *μ_a_* is the absorption coefficient in cm^−1^. The equation used for spectral unmixing, for each pixel, is therefore of the form:
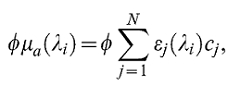
where *λ_i_* is the wavelength, *ϕμ_a_* is the optoacoustic response for that pixel, *ε_j_* is the wavelength-dependent absorption coefficient per concentration for the absorber represented by the spectral component *j*, and *c_j_* is the local concentration of that absorber. The unknown quantities solved for by least-squares are then *ϕc_j_* for *j = 1..N*. Note that the images must be corrected for possible wavelength-dependent fluctuations in laser excitation energy, so that *ϕ* is independent of wavelength. Additionally, the wavelength-dependence of the fluence due to the absorption spectrum of the tissue should either be corrected for or assumed to be negligible if sufficiently near to the skin surface.

An alternative approach to spectral decomposition in MSOT is blind unmixing [21]. In this work, Principal Component Analysis (PCA) was used to track the injected IRdye800-CW in the kidneys. PCA identifies the directions in multivariate data that represent maximum variance. A transform to these newly found basis vectors allows identification of different spectral signatures in the dataset. An advantage of this approach is that it does not require any *a priori* information regarding the absorbers present in the sample.

### Ratio normalization

In order to make a meaningful comparison of agent concentration across measurements spanning over a longer time period, we normalize the results from the unmixing procedure by the total signal strength per pixel, i.e.:
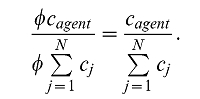



The result is a measure of the relative agent concentration per pixel, independent of differences in signal because of fluence variations due to attenuation inside the tissue or variations in laser energy over time.

### Fast MSOT animal imaging

We used a total of 10 adult CD1 mice, which were anaesthetized with 2% Isofluorane throughout the experiments. We chose 5 excitation wavelengths per experiment based on the maxima and minima in the absorption spectra of the imaging agents and tissue absorbers (ICG: 700 nm, 730 nm, 760 nm, 800 nm, 850 nm; CW800-COOH: 700 nm, 730 nm, 760 nm, 774 nm, 850 nm).

MSOT imaging was performed according to the following experimental protocol:

- Multispectral imaging prior to injection
- Imaging at peak agent absorption wavelength during injection
- Multispectral imaging post injection continuously for 20–30 minutes

Multispectral imaging was performed with 50 signal averages (laser pulses) per wavelength for liver and kidney imaging and 10 signal averages for circulation imaging to allow a more finely resolved time axis during the fast decay. We injected doses of 40 nmol for ICG and 20 nmol for CW800-COOH. The imaged slices were: a region in the either the lower abdomen or neck with visible blood vessels for the characterization of ICG in the circulation (3 mice), the liver at a slice where the gallbladder is also visible (3 mice), and a slice through the kidneys (4 mice).

### Validation by fluorescence cryoslicing

For validation of agent biodistribution, mice (total of 4) were injected in the same way as for the MSOT experiments and then euthanized by cervical dislocation at the relevant time points. The animals were then embedded in an optimal cutting temperature compound (Sakura Finetek Europe B. V., Zoeterwonde, NL) and frozen to −80°C. We then performed *ex vivo* validation using fluorescence cryoslicing imaging (FCSI) [22]. Similarly to the MSOT imaging geometry, FCSI sliced the frozen mice in the axial dimension, at a 500 µm micron pitch, and recorded color and fluorescence images from each slice. The FCSI system is based on a cryotome fitted with selectable excitation and emission filters and CCD-based detection. Fluorescence images were captured using a 785 nm longpass emission filter to resolve the biodistribution of the injected ICG and IRDye800-CW.

## Results

### Circulating ICG

**Figure 2:**
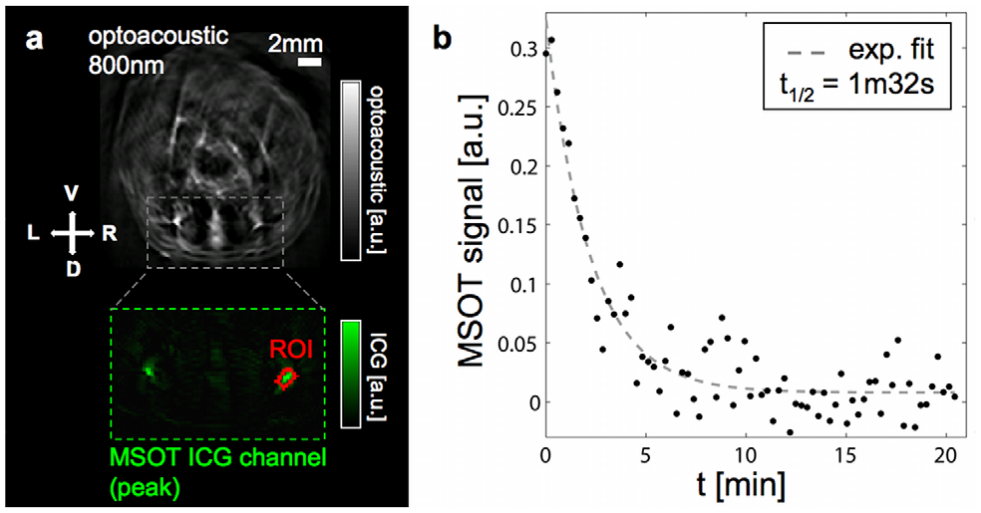
Imaging ICG in the circulation. a) Optoacoustic image of cross-sectional slice at 800 nm excitation (grayscale) and MSOT image of ICG signal (green) for a portion of the same slice. These images correspond to the first multispectral data point after injection. The selected ROI is outlined in red. b) Plot of the mean ICG signal value inside the ROI for each multispectral data point (black dots) and corresponding exponential decay fit (dashed line).

We performed *in vivo* imaging of a slice through the lower abdomen to characterize the circulation kinetics of ICG (Fig. 2). Several small blood vessels are visible in the MSOT images (Fig. 2a)—we selected a region of interest (ROI) corresponding to the ischiatic vein. After linear spectral unmixing for the absorption of ICG and subsequent ratio normalization, we plotted the mean value of the resulting signal amplitude inside the ROI (Fig. 2b). The time between two complete multispectral measurements (black dots) is approximately 17 s in this case. By a simple exponential decay fitting for the ICG disappearance rate (dashed line in Fig. 2b) we obtained a value for the circulation half-life measured in the selected blood vessel of 1 minute 32 seconds.

### Liver and gallbladder uptake of ICG

**Figure 3:**
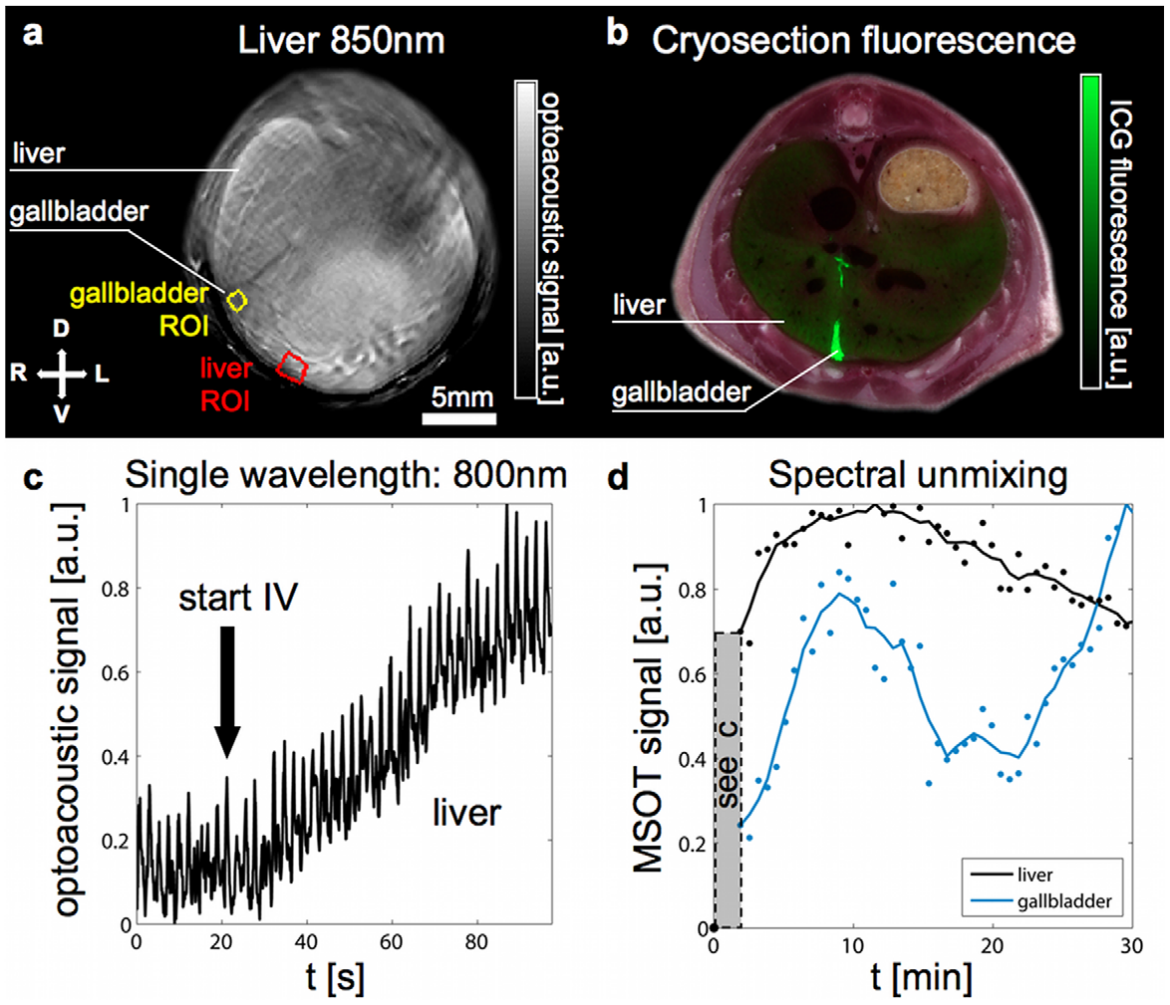
Liver and gallbladder uptake of ICG. a) Optoacoustic images through the liver. Grayscale image (left) showing anatomy and ROIs for liver (red) and gallbladder (yellow) analysis. b) FCSI image: fluorescence from ICG overlaid in green on color photograph of cryosection of a mouse sacrificed 10 minutes after injection, showing signal in the liver and gallbladder c) Plot of the signal increase in the liver ROI at 800 nm during single wavelength imaging of the ICG injection. Oscillations are mainly due to breathing motion. The scale is normalized to the maximum value. d) Specific (unmixed) signal from ICG after injection in the liver (black) and gallbladder (blue) ROIs. Each curve is normalized to its own maximum value.

ICG injected systemically binds to plasma proteins and is removed from the circulation by the liver. We imaged this uptake in the liver using two techniques: before, during and directly after injection we continuously acquired images at the full rate of 10 frames per second at 800 nm (near the absorption peak of ICG) to capture the initial rapid change in signal levels with maximum time resolution. Following this, we imaged multispectrally over a longer period of time (approximately 30 minutes) to capture further changes in the specific ICG signal. To extract a metric of relative ICG concentration, we selected two ROIs, one in the liver and one in the gallbladder (see outlines in Fig. 3a). Analysis of the liver ROI during and directly after injection yields a clear picture: the signal increases over its original level with increasing agent concentration (Fig. 3b). Note that the plotted data shows strong oscillations. These almost periodic spikes in the data correspond exactly to the breathing cycle of the mouse as verified by analysis of the captured image sequence. As the sampling rate is much higher than the breathing rate in this case, it is possible to smooth the data by neglecting those images where the mouse moves away from its normal position. For the multispectral data, the relative concentration measures obtained using linear spectral unmixing and ratio normalization show differing signal curves for the liver and gallbladder as their respective functions were visualized over a period of approximately 30 mins (Fig. 3d).

### Renal clearance of CW800-COOH

**Figure 4:**
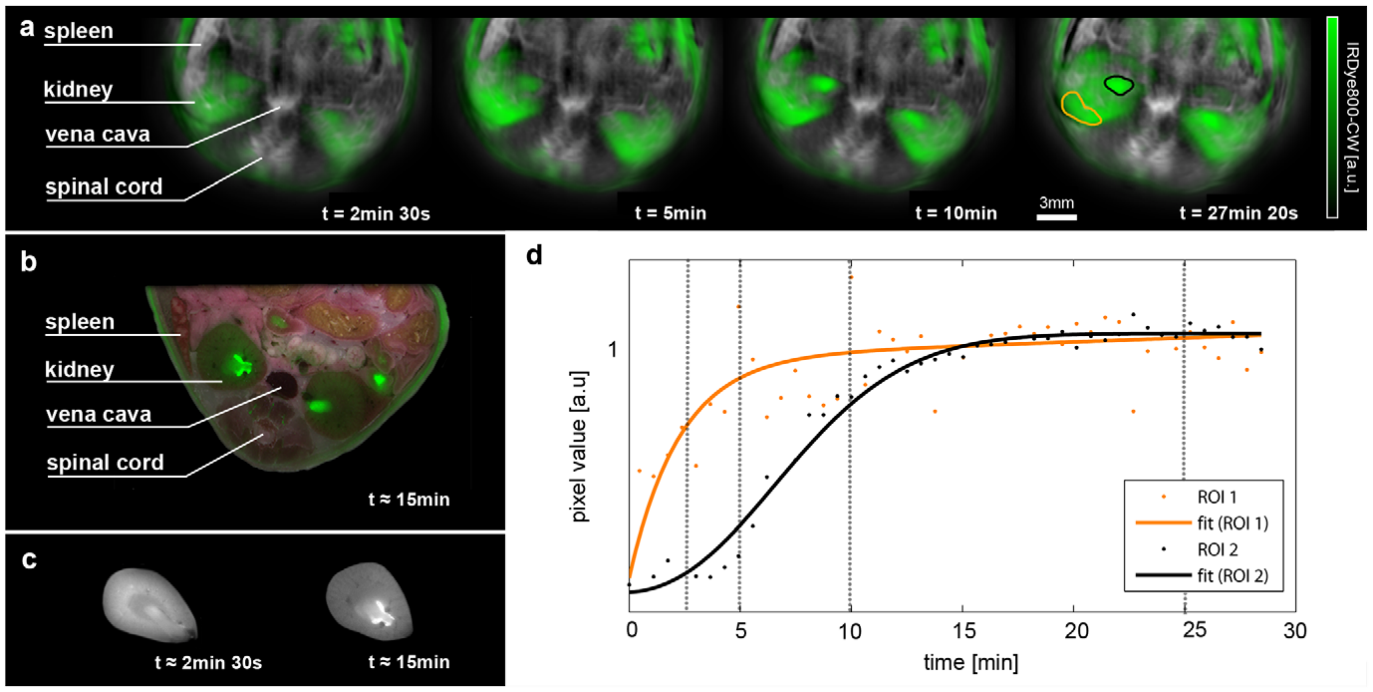
Kidney imaging. a) Time series of images visualizing the biodistribution of IRdye800 in green on logarithmic scale overlaid on the vasculature. Both channels are the result of spectral unmixing. b) Cryoslice image after approximately 15 minutes with overlaid fluorescence as a verification of the MSOT results. c) A comparison of fluorescence distribution in the kidneys of mice sacrificed after approximately 2 minutes 30 seconds after injection and 15 minutes after injection. Note the changes in distribution similar to the time series shown in a). d) Temporal evolution of signal (each normalized to their smoothed maxima) in the regions of interest highlighted in the rightmost image, orange showing a region in the renal cortex that displays early and steep signal pickup and black indicating a region in the renal pelvis where probe accumulation is delayed and has a smoother profile. Time points of the images in a) are marked using vertical lines.

Injected IRDye800-CW carboxylate is rapidly filtered by the kidneys. PCA unmixing revealed the biodistribution of the injected agent over time as shown in the green overlays in Fig. 4a. The grayscale background in Fig. 4a represents the component with an absorption spectrum corresponding to oxygenated hemoglobin, showing vascular structures. The imaged slice shows both kidneys. A representative cryosection with annotations is provided in Fig. 4b, a fluorescent overlay indicates the biodistribution of the dye 15 minutes after injection to validate the images produced by MSOT. Additionally, to validate the changes in distribution seen over time in the MSOT images, we compared the kidney fluorescence distribution in mice sacrificed after approximately 2 minutes 30 seconds and 15 minutes respectively (Fig. 4c). As in the MSOT images, the IRDye800-CW signal moves inwards towards the ureter over time. Note that the temporal resolution associated with sacrificing animals so soon after injection is not sufficient for more finely resolved *ex vivo* validation. This is because of the challenges involved in quickly halting all relevant physiological action, such as the heartbeat.

The images indicate different dynamics in different areas of the kidneys, where two regions of interest (ROI) have been highlighted in the last time point shown. The region corresponding to the renal cortex (Fig. 4a, orange), where filtration occurs, shows a fast pick-up in the signal curve after injection (Fig. 4d). Individual multispectral measurements acquired with a rate of approximately 2 per minute are shown as dots while the solid line represents a fit to an analytical function. The second ROI marked in black is located in the renal pelvis, where elimination towards the ureter takes place. Black dots and the respective fit (Fig. 4d) indicate a delayed and slower signal pick-up. The combination of these two curves clearly suggest that two dependent processes are being imaged, firstly the filtration of agent in the cortex and subsequently the excretion towards the ureter.

## Discussion

We have demonstrated MSOT as a tool for fast pharmacokinetics and biodistribution imaging of optical agents. The use of fluorescence in biomedical research is ubiquitous, ranging from fluorescent proteins through targeted agents to enzyme-activatable probes. Epi-illumination NIRF imaging is commonly used for biodistribution studies *in vivo*. However, such methods produce surface-weighted images where the high degree of photon scattering in tissue obstructs the true agent distribution, particularly in deeper organs. Tomographic approaches like fluorescence molecular tomography (FMT) solve this problem by producing quantitative 3D images in mice, but serial data collection results in long acquisition times of several tens of minutes per image [7]. In our implementation of MSOT, as demonstrated in the presented data, we are able to capture single wavelength images at 10 frames/s and multispectral data sets within seconds: in our experiment measuring ICG in the circulation, 1 s per wavelength was required for acquisition using 10 signal averages and approximately 3 s per wavelength change. This time could be further reduced: the OPO wavelength is tuned by mechanically scanning a crystal—faster scanning would reduce the acquisition time. The amount of signal averaging to use is a matter for further investigation. In our studies, signal averaging is primarily used to avoid excessive waiting times for wavelength changes. Ideally, one multispectral data point would be captured within such a short time that significant changes in agent concentration could not occur. Thereafter, multiple points could be binned or smoothed together to increase the detection sensitivity to the required level. A further application area for MSOT in biodistribution studies could be the imaging of non-fluorescent photo-absorbing materials, in particular those with absorption in the near-infrared, for example, gold nanorods [13] or carbon nanotubes [17].

The experimental MSOT implementation presented here does have limitations. Probably the most significant of these involves the 2-dimensional nature of the system—it is capable of imaging only 1 transverse slice at a time. While the animal holder can be translated to a different slice position automatically for imaging of different regions, doing this during experiments sacrifices temporal resolution in cases where more than one organ should be imaged simultaneously. Future development work in small animal MSOT imaging systems will aim to image a larger volume at a time, potentially solving this problem using more detector elements in a 3-dimensional arrangement. Regarding the use of the technique in larger animals, the primary limitation is the penetration depth of light.

One of the critical steps in the drug discovery process is the safety and toxicity evaluation of novel pharmaceuticals. As the major metabolic and excretory organs, liver and kidneys are of much concern in these trials. It is therefore highly important to develop a fast, noninvasive tool to assess liver and kidney function. MSOT can be used to not only determine the biodistribution of a multitude of injected agents, but it can be used in conjunction with organ-specific dyes such as ICG and IRDye800-CW to acquire a general assessment of organ function. Comparisons between the kinetics of ICG/IRDye800-CW clearance before and after drug treatment, for example, could be used to quickly assess organ function and drug-related acute toxicity. Clinical applications of high speed MSOT imaging are realistic: it is worth noting, for example, that the characterization measurements of ICG in the circulation could be performed on blood vessels in humans with minimal additional effort.
